# Differences in obesity-related health behaviors and health outcomes by rural and Appalachian residency

**DOI:** 10.1007/s10552-023-01741-8

**Published:** 2023-07-27

**Authors:** Xiaochen Zhang, Abigail B. Shoben, Ashley S. Felix, Brian C. Focht, Electra D. Paskett

**Affiliations:** 1 Public Health Sciences, Fred Hutchison Cancer Center, Seattle, WA USA; 2 Division of Cancer Prevention and Control, Department of Internal Medicine, College of Medicine, The Ohio State University, 1590 N High Street, Suite 525, Columbus, OH USA; 3 Division of Biostatistics, College of Public Health, The Ohio State University, Columbus, OH USA; 4 Division of Epidemiology, College of Public Health, The Ohio State University, Columbus, OH USA; 5 Kinesiology, Department of Human Sciences, The Ohio State University, Columbus, OH USA

**Keywords:** Obesity, Health behaviors, Health disparities, Rural, Appalachian

## Abstract

**Purpose:**

Obesity and health behaviors are the major modifiable contributors to cancer and health disparities. We examined the differences in obesity-related health behaviors, and health outcomes by rural and Appalachian residency in Ohio.

**Methods:**

Cross-sectional survey data from the 2011–2019 Behavioral Risk Factor Surveillance System were obtained from the Ohio Department of Health. County-level identifiers were used to classify urban non-Appalachian, urban Appalachian, rural non-Appalachian, and rural Appalachian residency. Self-reported weight, height, health behaviors, and health conditions were used. Logistic regression was used to assess the difference in health behaviors and health outcomes by rural and Appalachian residency. All analyses incorporated with sample weights.

**Results:**

Among Ohio residents, compared to urban non-Appalachian residents, urban Appalachian and rural Appalachian residents had a higher prevalence of obesity, hypertension, high cholesterol, and cardiovascular diseases, as well as lower rates of healthy diet and physical activity. No difference was found in trends of obesity and obesity-related health outcomes in 2011–2019 by rural and Appalachian residency. However, rural Appalachian residents had a greater increase in obesity, hypertension, and diabetes, whereas rural non-Appalachian had favorable changes in obesity-related health behaviors. Additionally, associations between health behaviors and obesity-related health outcomes differed by rural and Appalachian residency.

**Conclusions:**

Findings underscore the importance of distinguishing between urban non-Appalachian, urban Appalachian, rural non-Appalachian, and rural Appalachian populations when assessing health disparities. While the trends of obesity and obesity-related health outcomes did not differ, the association between health behaviors and obesity-related outcomes differed by rural and Appalachian residency.

**Supplementary Information:**

The online version contains supplementary material available at 10.1007/s10552-023-01741-8.

## Background

More than 30% adults in the United States (U.S) have overweight (body mass index: BMI = 25–29.9 kg/m^2^) and 42% have obesity (BMI ≥ 30 kg/m^2^) [1]. It is projected that by 2030, about one in two U.S. adults will have obesity [2]. Obesity is one of the leading preventable causes of cancer [3], with approximately 40% of cancer cases and 14–20% of cancer-specific deaths attributable to overweight and obesity [4]. Healthy behaviors, characterized by physical activity and a healthy diet can attenuate the negative effect of obesity, reduce cancer risk, and lower premature mortality [5, 6]. However, most U.S adults are physically inactive and have a poor diet [7, 8].

Residents in rural and Appalachian areas have higher rates of obesity, obesity-related comorbidities and mortality, as well as higher cancer incidence and mortality compared to residents in urban or non-Appalachian areas [9]. Socioeconomic and environmental disadvantages, including low income, lack of resources and availability related to health and health care, transportation barriers, geographic isolation, and lack of awareness and skills, could explain obesity-related disparities in Appalachian and rural areas [10−14]. Poor health behaviors, however, are the modifiable contributors to obesity-related disparities in rural and Appalachian populations [15–18]. Specifically, compared to urban residents, rural residents had higher rates of no leisure-time physical activity (42% vs. 33%), consumed ≥ 24 oz/day of sweetened beverage (56% vs. 46%), had < 1 cup/day of vegetable (36% vs. 32%), and had < 1 cup/day of fruit (73% vs. 62%) [17, 18]. Similarly, Appalachian residents are more likely to be physically inactive compared to national estimates (28.4% vs. 23.1%) [16].

However, Appalachian areas are not all rural. Within the Appalachian region, 42% of the population is rural, compared to 20% of the population in the U.S. [19]. Compared to residents from the large metro Appalachian areas, higher percentages of rural Appalachian residents were obese, diagnosed with diabetes, and physically inactive [16]. Unique personal and social factors in Appalachian populations, including unfavorable beliefs about health behaviors, unhealthy social norms, and familial influences, may explain the variations of health behaviors and obesity-related disparities from their non-Appalachian counterparts [20–22]. When examining obesity-related health disparities, most studies distinguish between rural vs. urban counties or Appalachian vs. non-Appalachian counties. Thus, the objective of this study was to identify the differences in obesity-related behaviors and health outcomes by rural and Appalachian residency among Ohio adults. We hypothesized that compared to urban non-Appalachian residents, rural Appalachian residents had poorer health behaviors and experienced greater burdens in obesity-related health outcomes. Findings from this study could facilitate the development of tailored interventions to improve health behaviors, reduce cancer risk, and address obesity-related health disparities in different populations.

## Methods

### Study sample

The 2011–2019 Behavioral Risk Factor Surveillance System (BRFSS) data were obtained through the Ohio Department of Health. The BRFSS is an ongoing random-digit-dialed telephone-based cross-sectional survey conducted by the Centers for Disease Control and Prevention (CDC) designed to measure behavioral risk factors in the U.S. adult population (≥ 18 years old) [23]. A total of 108,740 Ohio residents completed the BRFSS survey from 2011 to 2019 [24]. This study was exempt from The Ohio State University Institutional Review Board review.

### Measures

Demographic characteristics (e.g., age, sex, race, ethnicity, marital status, education, employment, household income) were self-reported. County of residency was identified using county-level Federal Information Processing Standard. The Rural–Urban Continuum Codes (RUCC, range 1–9) was used to define rural vs. urban counties [25]. Counties with a score of 1–3 were classified as metropolitan (urban), and a score of 4–9 were classified as nonmetropolitan (rural). Based on this definition, 50 of 88 Ohio counties are rural. According to the Appalachian Regional Commission, 32 of the 88 counties are designated as Appalachian counties [9]. To account for both rural/urban and Appalachian/non-Appalachian classifications, our study classified Ohio counties as urban non-Appalachian (*n* = 28), urban Appalachian (*n* = 10), rural non-Appalachian (*n* = 28), or rural Appalachian (*n* = 22).

Self-reported body weight and height were used to calculate body mass index (BMI, kg/m^2^) and classify participants as whether they had obesity (BMI ≥ 30 kg/m^2^). Obesity-related health outcomes were self-reported based on the positive affirmation of any of the following conditions: “Has a doctor, nurse, or other health professionals ever told you that you had high blood pressure/ high blood cholesterol/ heart attack/ coronary heart disease/ stroke/ any types of cancer (other than skin cancer)/ diabetes?”.

Questions regarding health behaviors varied across survey years (Online Resource SI Table 1). All participants were asked whether they participated in any physical activities other than their regular job during the past month. Participants from the year of 2011, 2013, 2015–2017, and 2019 were asked about the types, frequency, and duration of the physical activity, and the frequency of strength training they participated. Participants were classified as whether they met aerobic physical activity guidelines (≥ 150 min/week moderate-vigorous physical activity), muscle-strengthening guidelines (at least 2 times/week), and both aerobic and strengthening guidelines [26, 27].

**Table 1:** Participants characteristics by rural and Appalachian residency, BRFSS 2011–2019 from Ohio Department of Health

Variable	Overall	Urban non-Appalachian	Urban Appalachian	Rural non-Appalachian	Rural Appalachian
	*N* = 84,762	*n* = 46,239 (69.11%)	*n* = 10,154 (8.83%)	*n* = 13,687 (12.28%)	*n* = 14,682 (9.77%)
Age at survey, years (mean ± SD)	49.98 ± 18.03	49.68 ± 17.92	50.61 ± 17.73	51.12 ± 17.74	50.09 ± 17.59
Sex					
Male	49.55%	49.03%	49.84%	50.67%	51.58%
Female	50.45%	50.97%	50.16%	49.33%	48.42%
Race/ethnicity					
Non-Hispanic white	82.09%	77.59%	89.28%	92.82%	93.92%
Non-Hispanic black	11.43%	15.16%	6.05%	1.75%	2.10%
Asian	1.24%	1.56%	0.62%	0.54%	0.40%
American Indian, Native Hawaiian, or Pacific islander	0.61%	0.66%	0.36%	0.40%	0.70%
Hispanic	2.36%	2.57%	1.87%	2.57%	1.11%
Other	2.27%	2.46%	1.82%	1.91%	1.76%
Marital status					
Married/unmarried couple	56.66%	54.96%	57.19%	63.13%	60.00%
Divorced/widowed/separated	22.76%	22.63%	23.88%	21.49%	24.21%
Never married	20.24%	22.01%	18.70%	15.11%	15.52%
Household income					
< $25K	25.36%	24.66%	27.34%	24.17%	30.05%
$25–49.9K	23.69%	22.86%	25.64%	25.98%	24.93%
$50–74.9K	14.51%	14.16%	14.70%	16.71%	14.07%
≥ $75K	24.38%	26.49%	20.41%	20.50%	17.95%
Educational level					
≤ Some high school	11.37%	10.47%	12.57%	11.71%	16.19%
High school graduate	34.20%	30.93%	39.72%	43.02%	41.29%
Some college/ technical school	30.76%	31.47%	29.47%	29.07%	29.00%
≥ College graduate	23.58%	27.03%	18.14%	16.09%	13.43%
Employment					
Employed	49.54%	50.78%	46.26%	48.86%	44.55%
Self-employed	7.20%	7.01%	7.31%	7.63%	7.97%
Out of work	5.93%	5.96%	6.63%	5.19%	6.00%
Homemaker/A Student	8.48%	8.49%	9.02%	7.66%	8.98%
Retired	20.67%	20.10%	21.14%	22.99%	21.37%
Unable to work	7.85%	7.34%	9.21%	7.45%	10.71%
BMI, kg/m^2^	28.45 ± 6.64	28.32 ± 6.57	28.65 ± 6.53	28.76 ± 6.62	28.78 ± 6.60

Participants from 2011 to 2013, 2015, 2017, and 2019 were asked their fruit and vegetable consumption (e.g., pure fruit juice, fruits, beans, dark green vegetables, orange-colored vegetables). Participants were classified as meeting fruit and vegetable recommendation if they self-reported consuming fruits and vegetables ≥ 4.5 times per day. Participants from 2013, 2015–2019 were asked about the consumption of sugary drinks (e.g., regular soda, sugar-sweetened fruit juice). Participants were classified as meeting sugary drink recommendations if they self-reported consuming sugary drinks ≤ three times per week.

### Statistical analysis

All analyses incorporated survey weights to represent the non-institutionalized Ohio population [24]. Participants who were < 20 years old, pregnant at the time of the survey, refused or had missing data on weight, county of residency, age, or sex were excluded from this analysis.

Descriptive statistics of the selected variables of interest were reported with appropriate survey weighting. For continuous variables, population-weighted means and standard deviations were reported and Analysis of Variance (ANOVA) was used to compare the differences by rural and Appalachian residency. For categorical variables, contingency tables with the variables of interest were constructed, and a chi-square test was used to compare differences by rural and Appalachian residency.

Multivariable logistic regression were used to estimate the prevalence of each outcome (obesity, obesity-related health behaviors, and health outcomes) by rural and Appalachian residency when controlling for age and sex [28]. To assess whether the trends of obesity, obesity-related behaviors and health outcomes differed by rural and Appalachian residency, the significance of interaction terms between rural and Appalachian residency and survey years were examined using logistic models. To determine whether the associations of health behaviors, obesity, and obesity-related health outcomes differed by rural and Appalachian residency, multivariable logistic regression models were constructed to estimate odds ratios (ORs) and 95% Confidence Intervals (CIs). The significance of interaction terms between rural and Appalachian residency and health behaviors from the logistic models were assessed to determine if the association differed by rural and Appalachian residency. Statistical tests were conducted with significance set at *p* < 0.05, whereas the significance of interaction was set at *p* < 0.10 [29]. This study included various statistical tests without adjusting for multiple comparisons. Adjustment for multiple comparisons is designed to avoid potentially spurious findings to maintain strict type I error control. However, in exploratory studies, strict type I error control decrease power for associations that are not null, which could miss possibly important findings worth of future investigation [30]. The primary focus for the analyses reported here was differences in obesity-related health behaviors and outcomes by rural and Appalachian residency since few studies had previously done so. All other analyses were exploratory. All analyses were completed between November 2021 and May 2022 using STATA IC 17.1 (StataCorp LLC, College Station, TX).

## Results

### Participant characteristics

The 2011–2019 Ohio BRFSS data included 105,825 adults who were 20 years or older. Among them, a total of 84,762 participants were identified with non-missing information regarding county of residency, sex, and BMI, thus were included in the analysis. Compared to those who had complete data, those who had missing data for county of residency, sex, and BMI (*n* = 21,064) were younger, less likely to be non-Hispanic White, had a household income < $25 K, had some high school or lower education, and were a homemaker/student or retired, and more likely to be never married (Online Resource SI Table 2, all *p* < 0.001).

**Table 2:** Predicted prevalence of obesity, obesity-related behaviors and health outcomes among Ohio adults (adjusting for age and sex), by rural and Appalachian residency, BRFSS 2011–2019 from Ohio Department of Health

Obesity-related outcome	Urban non-Appalachian	Urban Appalachian	Rural non-Appalachian	Rural Appalachian	*p* value
	*n* = 46,239 (69.11%)	*n* = 10,154 (8.83%)	*n* = 13,687 (12.28%)	*n* = 14,682 (9.77%)	
Obesity^a^	31.94%	34.17%	34.71%	34.35%	< 0.001
Health outcomes					
Hypertension	35.26%	37.86%	35.49%	37.95%	0.002
High cholesterol	35.82%	38.30%	34.68%	38.33%	0.001
Diabetes	12.59%	13.14%	12.54%	13.39%	0.183
Cancer	7.30%	7.94%	7.17%	7.86%	0.119
Any CVD	9.86%	10.79%	10.39%	11.99%	< 0.001
Myocardial infarction	5.34%	6.51%	6.05%	7.02%	< 0.001
Coronary heart disease	4.93%	5.89%	5.43%	6.37%	< 0.001
Stroke	3.80%	3.49%	3.71%	4.38%	0.045
Dietary intake					
Consumed ≥ 1 veggie/day	77.69%	75.07%	76.26%	75.36%	0.007
Consumed ≥ 1 fruit/day	60.38%	56.33%	56.79%	55.28%	< 0.001
Met fruit and veggie guideline^b^	3.95%	3.16%	4.26%	3.16%	0.026
Met sugary drink guideline^c^	18.85%	16.57%	14.03%	13.97%	< 0.001
Physical activity					
Any exercise	73.73%	70.11%	70.25%	68.06%	< 0.001
Met aerobic guideline^d^	52.77%	51.69%	49.42%	50.72%	0.003
Met strengthening guideline^e^	31.94%	29.89%	26.72%	25.67%	< 0.001
Met both PA guidelines	22.10%	21.40%	17.51%	18.57%	< 0.001

^a^BMI ≥ 30 kg/m^2^

^b^Fruit and veggetable guideline: ≥ 4.5 times/day

^c^Sugary drink recommendation: ≤ 3 drinks/week

^d^Aerobic guideline: ≥ 150 min/week moderate-to-vigorous intensity physical activity

^e^Strengthening guideline: at least 2 times/week

Among the included participants, weighted mean age at completing the BRFSS survey was 50.0 ± 18.0 years, and weighted mean BMI was 28.5 ± 6.6 kg/m^2^ (Table 1). Half of the participants were female, and most were non-Hispanic White (82.1%). Participant characteristics differed by rural Appalachian residency. Urban non-Appalachian participants were younger, more likely to be female, had a household income $75 K or more, employed, and less likely to be non-Hispanic White and married or a member of an unmarried couple. Whereas rural Appalachian participants were more likely to be male, non-Hispanic White, had a household income less than $25 K, had lower than high school education, and a higher BMI.

After accounting for age and sex, the predicted prevalence of obesity (BMI ≥ 30 kg/m^2^) between 2011 and 2019 was highest among rural non-Appalachian participants (Table 2, 34.7%), followed by rural Appalachian (34.4%) and urban Appalachian (34.2%), and lowest among urban non-Appalachian participants (31.9%). Compared to urban non-Appalachian participants, participants from urban Appalachian and rural Appalachian areas had a higher prevalence of hypertension, high cholesterol, and any cardiovascular disease (all *p* < 0.05). The prevalence of diabetes and cancer did not differ by rural Appalachian residency.

In terms of health behaviors, compared to urban non-Appalachian counterparts, urban Appalachian and rural Appalachian participants had lower rates of consuming ≥ 1 vegetable/day (Table 2, 75.1% and 75.4%, vs. 77.7%, *p* = 0.01) and ≥ 1 fruit/day (56.3% and 55.3%, vs. 60.4%, *p* < 0.001). The rate of meeting the sugary drink recommendation (≤ 3 times/week) was highest among urban non-Appalachian residents (18.9%), followed by urban Appalachian (16.6%), rural non-Appalachian (14.0%), and lowest in rural Appalachian residents (14.0%, *p* < 0.001). Similarly, rates of participating in any exercise and meeting strengthening guidelines were highest among urban non-Appalachian participants (73.7% and 31.9%) and lowest among rural Appalachian participants (68.1% and 25.7%). While urban non-Appalachian participants had highest rate of meeting aerobic guidelines (≥ 150 min/week moderate-vigorous intensity physical activity, 52.8%), rural Appalachian participants had higher rates (50.7%) compared to rural non-Appalachian participants (49.4%).

**Fig. 1:**
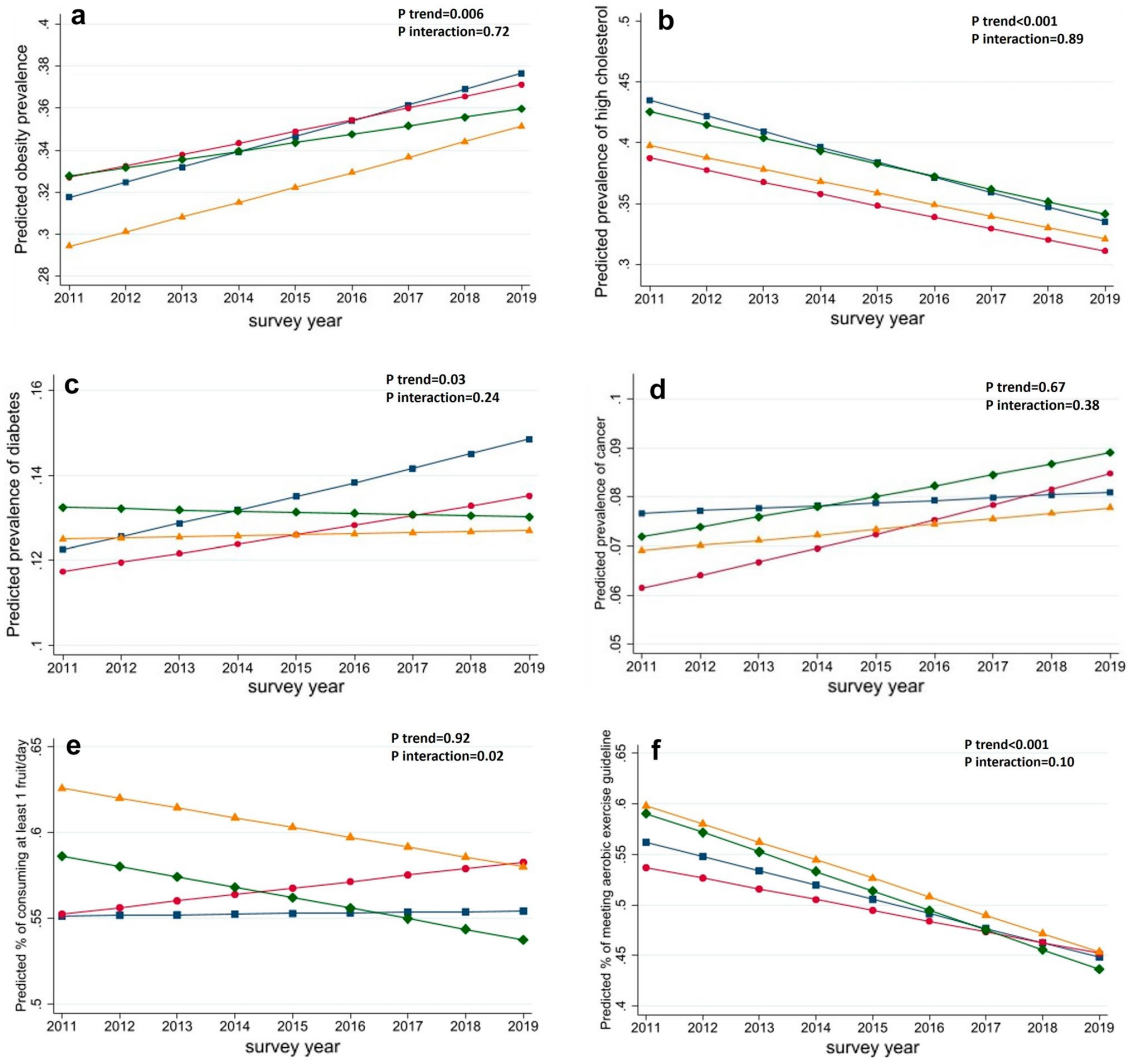
Trends of obesity, obesity-related behaviors and health outcomes among Ohio adults by rural and Appalachian residency in 2011–2019 (**a** Obesity, **b** High Cholesterol, **c** Diabetes, **d** cancer, **e** Consumed ≥ 1 fruit/day, **f** Met aerobic exercise guideline)

The trends of obesity and obesity-related health outcomes in 2011–2019 did not differ by rural and Appalachian residency (Online Resource SI Table 3). Regardless of rural and Appalachian residency, the prevalence of obesity (*p* trend = 0.006) and diabetes (*p* trend = 0.03) increased across the years, while the prevalence of high cholesterol (*p* trend < 0.001) decreased over time (Fig. 1a–c). However, the prevalence of cancer did not change over time (Fig. 1d, *p* trend = 0.67). The trend of consuming at least one serving of fruits per day and meeting aerobic exercise guidelines differed between urban Appalachian, urban non-Appalachian, rural Appalachian, and rural non-Appalachian residents (Online Resource SI Table 3). Specifically, between 2011 and 2019, the percentage of participants who consumed ≥ 1 serving of fruits per day decreased among urban non-Appalachian and urban Appalachian residents and increased among rural Appalachian and rural non-Appalachian residents (insert Fig. 1d) (*p*_interaction_ = 0.02, Fig. 1e). While the percentage of participants who met aerobic exercise guidelines decreased over time, the magnitude of the decrease was greatest among urban Appalachian residents, followed by urban non-Appalachian, rural Appalachian, and smallest among rural non-Appalachian residents (insert Fig. 1e) (*p*_interaction_ = 0.10, Fig. 1f).

**Table 3:** The association between health behaviors and health outcomes among Ohio adults by rural and Appalachian residency, BRFSS 2011–2019 from Ohio Department of Health

Outcome exposure (yes vs. no)	Urban non-Appalachian	Urban Appalachian	Rural non-Appalachian	Rural Appalachian	Health behavior by rural/Appalachian
	*n* = 46,239	*n* = 10,154	*n* = 13,687	*n* = 14,682	*p*_interaction_
	OR (95% CI)	OR (95% CI)	OR (95% CI)	OR (95% CI)	
Obesity					
Any exercise	0.57 (0.53, 0.60)	0.65 (0.56, 0.75)	0.65 (0.57, 0.73)	0.67 (0.59, 0.75)	0.037
Aerobic guideline	0.61 (0.56, 0.66)	0.66 (0.54, 0.80)	0.67 (0.58, 0.78)	0.65 (0.55, 0.75)	0.618
Strength guideline	0.62 (0.57, 0.68)	0.66 (0.53, 0.82)	0.67 (0.57, 0.80)	0.57 (0.47, 0.69)	0.605
Fruit veg guidelines	0.61 (0.49, 0.74)	0.70 (0.43, 1.15)	0.63 (0.43, 0.93)	0.81 (0.55, 1.20)	0.601
Sugary drink	0.89 (0.74, 1.08)	0.58 (0.35, 0.95)	0.94 (0.62, 1.41)	1.40 (0.99, 1.98)	0.029
Diabetes					
Any exercise	0.62 (0.57, 0.67)	0.74 (0.62, 0.88)	0.67 (0.58, 0.77)	0.73 (0.64, 0.84)	0.083
Aerobic guideline	0.67 (0.60, 0.74)	0.61 (0.48, 0.78)	0.70 (0.58, 0.85)	0.68 (0.57, 0.81)	0.845
Strength guideline	0.68 (0.60, 0.76)	0.65 (0.49, 0.84)	0.71 (0.57, 0.89)	0.78 (0.63, 0.98)	0.627
Fruit veg guidelines	1.12 (0.84, 1.49)	0.97 (0.57, 1.65)	0.79 (0.47, 1.33)	1.10 (0.73, 1.66)	0.694
Sugary drink	1.17 (0.85, 1.61)	0.58 (0.24, 1.40)	0.62 (0.37, 1.04)	1.53 (0.95, 2.46)	0.035
Any CVD					
Any exercise	0.67 (0.61, 0.73)	0.72 (0.59, 0.87)	0.73 (0.62, 0.86)	0.84 (0.72, 0.99)	0.101
Aerobic guideline	0.74 (0.67, 0.84)	0.85 (0.66, 1.10)	0.62 (0.51, 0.76)	0.86 (0.70, 1.05)	0.121
Strength guideline	0.88 (0.78, 1.00)	0.71 (0.53, 0.95)	0.91 (0.72, 1.16)	1.07 (0.83, 1.37)	0.212
Fruit veg guidelines	0.87 (0.63, 1.20)	1.29 (0.67, 2.48)	1.01 (0.55, 1.87)	1.31 (0.79, 2.16)	0.509
Sugary drink	0.74 (0.54, 1.01)	1.47 (0.60, 3.58)	0.95 (0.52, 1.73)	0.97 (0.62, 1.53)	0.441
Cancer					
Any exercise	0.90 (0.82, 0.99)	1.21 (0.97, 1.51)	0.91 (0.76, 1.09)	1.00 (0.84, 1.19)	0.086
Aerobic guideline	0.99 (0.88, 1.11)	1.23 (0.92, 1.64)	1.02 (0.82, 1.27)	1.01 (0.81, 1.27)	0.582
Strength guideline	0.90 (0.79, 1.04)	1.11 (0.81, 1.52)	1.08 (0.84, 1.38)	1.11 (0.87, 1.42)	0.315
Fruit veg guidelines	1.33 (1.01, 1.74)	1.02 (0.48, 2.15)	0.86 (0.50, 1.49)	0.76 (0.47, 1.24)	0.188
Sugary drink	0.93 (0.68, 1.26)	0.71 (0.28, 1.80)	0.59 (0.32, 1.11)	0.94 (0.57, 1.54)	0.607

In terms of the association of health behaviors with obesity and obesity-related health outcomes, using a significance level of *p* = 0.10 for the interaction term, we observed some differences by rural and Appalachian residency. When comparing those who reported any exercise vs. no exercise, the odds of obesity was 43% lower among urban non-Appalachian residents, 35% lower among urban Appalachian and rural non-Appalachian, and 33% lower among rural Appalachian residents (Table 3, *p*_interaction_ = 0.04). The odds of diabetes was lowest among urban non-Appalachian, followed by rural non-Appalachian, rural Appalachian, and urban Appalachian residents (*p*_interaction_ = 0.08). The odds of any CVD was lowest among urban non-Appalachian, followed by urban Appalachian, rural non-Appalachian, and rural Appalachian residents (*p*_interaction_ = 0.10). The association of lower odds of cancer and any exercise was only observed among urban non-Appalachian residents (*p*_interaction_ = 0.09).

Comparing those who met sugary drink guidelines to those not meeting the guidelines, the odds of obesity was 42% lower among urban Appalachian residents, although no association was observed among urban non-Appalachian, rural non-Appalachian, or rural Appalachian residents (*p*_interaction_ = 0.03). Interestingly, meeting the sugary drink guidelines was associated with lower odds of diabetes among urban Appalachian and rural non-Appalachian residents, but increased odds among urban non-Appalachian and rural Appalachian residents (*p*_interaction_ = 0.04). All other interactions were not significant.

## Discussion

This study examined obesity-related health behaviors and outcomes by rural and Appalachian residency, and identified three key findings: (1) compared to urban non-Appalachian participants, urban Appalachian and rural Appalachian participants had a higher prevalence of obesity and obesity-related diseases, with lower rates of healthy behaviors; (2) trends of obesity and obesity-related health outcomes between 2011 and 2019 did not differ by rural and Appalachian residency; and (3) the association between health behaviors and obesity-related health outcomes differed by rural and Appalachian residency. When discussing health disparities in underserved geographic areas, previous research often reported differences by rural/urban [31, 32] or Appalachian/non-Appalachian status [15, 16]. Our study shows that comparing rural/urban or Appalachian/non-Appalachian alone precludes the identification of crucial obesity-related health disparities among these four groups.

We found that, in state of Ohio, rural Appalachian residents reported the highest prevalence of hypertension, high cholesterol, and cardiovascular diseases, with the lowest rates of consuming more than one fruit per day, meeting guidelines for fruit and vegetable intake, sugary drinks, strengthening exercise, and participating in any exercise. However, urban Appalachian residents had the lowest rates of consuming ≥ 1 serving of vegetables and meeting fruit & vegetable guidelines, whereas rural non-Appalachian residents had the lowest rates of meeting national physical activity guidelines. These findings suggest the need to address the variation of obesity-related health disparities among urban Appalachian, rural non-Appalachian, and rural Appalachian residents. For example, management of obesity-related health conditions may be more warranted among rural Appalachian populations, while promotion of physical activity and increased fruit and vegetable intake may be higher priorities for rural non-Appalachian and urban Appalachian residents.

Another aspect of this study was to examine trends of obesity-related health behaviors and outcomes over time by rural and Appalachian residency. The socioeconomic, personal, and environmental barriers, including geographic isolation, societal beliefs, low health literacy, and fatalistic beliefs about health, all contribute to unfavorable health behaviors and outcomes in rural and Appalachian populations [20–22]. Several health promotion programs, such as health education, faith-based interventions, and community-based programs, have been implemented in both rural and Appalachian areas in the last decades [33–35]. Between 2011 and 2019, Ohio residents from rural non-Appalachian reported favorable changes in some obesity-related health behaviors (e.g. consuming at least one fruit per day). Despite the fact that some progress has been made in terms of healthy behaviors, obesity-related health disparities persist, and in some cases expanded, in rural Appalachian populations. In our study, rural Appalachian residents reported a greater increase in the prevalence of obesity, hypertension, and diabetes. These are the same populations who are more likely to have limited access to healthcare, lower health literacy, and delayed disease diagnosis, resulting in severe complications (e.g., lower limb amputation, depression, macrovascular and microvascular consequences) [36]. It is no coincidence that the increased prevalence of obesity, diabetes, and hypertension in rural Appalachian areas overlaps with health care professional shortages [37]. To address obesity-related health disparities, improving access to healthcare and attenuating shortages of healthcare professionals are critical, especially for the rural Appalachian population.

Furthermore, we demonstrated that associations between obesity-related health behaviors and outcomes differed by rural and Appalachian residency. For instance, participating in any exercise was associated with lower odds of obesity and cardiovascular diseases. The magnitude of the decreased odds was greater in urban non-Appalachian residents compared to rural Appalachian residents. Similarly, meeting sugary drink guidelines was associated with lower odds of obesity, hypertension, and diabetes. The magnitude of the decreased odds was greater among urban Appalachian populations. Due to the cross-sectional survey design and self-reported data, we were not able to investigate the underlying mechanisms that cause these variations by rural/Appalachian residency. However, one-size-fits-all approaches to address obesity-related health outcomes may not be effective across urban non-Appalachian, urban Appalachian, rural non-Appalachian, and rural Appalachian populations. Conversely, our findings highlight the importance of designing and implementing tailored interventions with behavioral components tailored for each population in order to achieve specific health benefits. For example, among urban non-Appalachian populations, it is reasonable to suggest interventions emphasize increasing physical activity; and for urban Appalachian populations, programs could be focused on reducing sugary drink consumption.

Our findings, however, are subject to several limitations. First, the analyses were based on self-reported data which may be affected by recall bias. Participants may overestimate physical activity level and fruit and vegetable intake. Residents from rural and Appalachian areas might underestimate their obesity-related health conditions due to lower education, lack of health awareness, and less access to healthcare, which may account for the differences in rural and Appalachian being less pronounced than in urban non-Appalachian areas [38]. Second, studies have demonstrated that response rates are lower in under-represented populations (e.g., racial/ethnic minorities, women, and younger individuals) [39, 40]. Although BRFSS utilizes complex sampling weights to account for selection probabilities and non-coverage, nonresponse bias in rural and Appalachian residents could exist. Third, this study included various statistical tests without adjusting for multiple comparisons. Additional studies are needed to confirm the statistical significance of our exploratory findings. Lastly, the cross-sectional study design limited our ability to infer the causality of the observed association between obesity-related health behaviors and health outcomes.

Strengths of this study include the geographic diversity of Ohio that allowed us to distinguish the differences in obesity-related health behaviors and outcomes among residents from urban non-Appalachian, urban Appalachian, rural non-Appalachian, and rural Appalachian areas. Using the county-level identifiers obtained from the Ohio Department of Health, we classified participants’ rural and Appalachian residency according to the RUCC, which minimizes the effects of variations in county size [25]. The BRFSS annual data and large sample size provided the opportunity to examine the differences in trends and determine interaction effects.

Our study underscores the importance of distinguishing urban non-Appalachian, urban Appalachian, rural non-Appalachian, and rural Appalachian populations when assessing health disparities within rural and Appalachian communities. Obesity-related health disparities persist, especially among rural Appalachian population. It is essential to implement strategies tailored to each population to address their unique needs, with the goal of reducing obesity-related disparities in rural and Appalachian areas.

### Supplementary Information

Below is the link to the electronic supplementary material.

Supplementary file1 (DOCX 23 kb)

## Data Availability

BRFSS data are publicly available at https://www.cdc.gov/brfss/index.html. For the state of Ohio BRFSS data, please contact the Ohio Department of Health to request access https://odh.ohio.gov/know-our-programs/behavioral-risk-factor-surveillance-system
