# Supplementary material for: Differences in obesity-related health behaviors and health outcomes by rural and Appalachian residency
Source: Cancer Causes Control. 2023 Jul 27;34(12):1113–21. doi: 10.1007/s10552-023-01741-8 (PMC10547622; doi:10.1007/s10552-023-01741-8)
Supplement: Supplementary file 1 — Supplementary file1 (DOCX 23 kb) [file 10552_2023_1741_MOESM1_ESM.docx]

SI Table 1. Differences of health behaviors assessed in Ohio BRFSS data from 2011-2019.

|  | 2011 | 2012 | 2013 | 2014 | 2015 | 2016 | 2017 | 2018 | 2019 |
| --- | --- | --- | --- | --- | --- | --- | --- | --- | --- |
| Any physical activity | X | X | X | X | X | X | X | X | X |
| Physical activity type, frequency, and duration | X |  | X |  | X | X | X |  | X |
| Frequency of strength training | X |  | X |  | X | X | X |  | X |
| Fruit & vegetable consumptions | X | X | X |  | X |  | X |  | X |
| Sugary drink |  |  | X |  | X | X | X | X | X |

SI Table 2. Characteristics between complete cases vs. incomplete cases, BRFSS 2011-2019 from Ohio Department of Health

|  |  | **Eligible participants^a^** | **Complete cases** | **Incomplete cases^b^** | P |
| --- | --- | --- | --- | --- | --- |
|  |  | n=105,825 | n=84,762 | n=21,064 |  |
| Age, yrs | | 49.52±0.08 | 49.98±0.09 | 47.75±0.18 | <0.001 |
| **Race/ethnicity** | |  |  |  | <0.001 |
|  | Non-Hispanic white | 81.2% | 82.1% | 78.0% |  |
|  | Non-Hispanic black | 11.4% | 11.4% | 11.1% |  |
|  | Asian | 1.6% | 1.2% | 2.8% |  |
|  | American Indian, Native Hawaiian, or pacific islander | 0.6% | 0.6% | 0.6% |  |
|  | Hispanic | 2.6% | 2.4% | 3.6% |  |
|  | Other | 2.6% | 2.3% | 3.9% |  |
| **Marital status** | |  |  |  | <0.001 |
|  | Married/a member of an unmarried couple | 56.2% | 56.7% | 54.3% |  |
|  | Divorced/widowed/ separated | 22.4% | 22.8% | 20.9% |  |
|  | Never married | 21.0% | 20.2% | 23.7% |  |
|  | Missing/refused | n=563 | n=303 | n=260 |  |
| **Household income** | |  |  |  | <0.001 |
|  | <$25K | 24.7% | 25.4% | 22.1% |  |
|  | $25-49.9K | 23.0% | 23.7% | 20.4% |  |
|  | $50-74.9K | 14.0% | 14.5% | 12.3% |  |
|  | ≥$75K | 24.3% | 24.4% | 24.1% |  |
|  | Missing/refused | n=16664 | n=11575 | n=5089 |  |
| **Educational level** | |  |  |  | <0.001 |
|  | ≤ Some high school | 11.3% | 11.4% | 10.8% |  |
|  | High school graduate | 33.5% | 34.2% | 31.0% |  |
|  | Some college/ technical school | 30.6% | 30.8% | 30.2% |  |
|  | ≥ College graduate | 24.3% | 23.6% | 26.9% |  |
|  | Missing/refused | n=354 | n=104 | n=250 |  |
| **Employment** | |  |  |  | <0.001 |
|  | Employed | 49.8% | 49.5% | 50.7% |  |
|  | Self-employed | 7.1% | 7.2% | 6.9% |  |
|  | Out of work | 5.8% | 5.9% | 5.1% |  |
|  | Homemaker/A Student | 8.9% | 8.5% | 10.4% |  |
|  | Retired | 20.2% | 20.7% | 18.4% |  |
|  | Unable to work | 7.7% | 7.9% | 7.3% |  |
|  | Missing/refused | n=505 | n=290 | n=215 |  |

Total population from BRFSS 2011-2019, N=108,785

^a^age>=20, not pregnant, n=105,826

^b^Missing BMI (n=7484), rural/appa (n=4700), sex (n=12,764)

SI Table 3. Differences in trends of obesity, obesity-related health behaviors and health outcomes among Ohio adults by rural and Appalachian residency in 2011-2019.

| Outcomes | Urban non-Appalachian | Urban Appalachian | Rural non-Appalachian | Rural Appalachian | Rural/Appalachian by survey year |
| --- | --- | --- | --- | --- | --- |
|  | OR (95% CI) | OR (95% CI) | OR (95% CI) | OR (95% CI) | P*_interaction_* |
| **Obesity**^a^ | 1.00 (0.98, 1.03) | 0.98 (0.95, 1.02) | 0.99 (0.96, 1.02) | ref | 0.720 |
| **Health Outcomes** |  |  |  |  |  |
| Hypertension | 1.00 (0.97, 1.03) | 0.99 (0.95, 1.04) | 0.99 (0.95, 1.03) | ref | 0.950 |
| High cholesterol | 1.01 (0.98, 1.04) | 1.01 (0.97, 1.05) | 1.01 (0.98, 1.05) | ref | 0.876 |
| Diabetes | 0.97 (0.94, 1.00) | 0.97 (0.93, 1.01) | 0.99 (0.96, 1.03) | ref | 0.237 |
| Cancer | 1.01 (0.97, 1.05) | 1.02 (0.97, 1.08) | 1.04 (0.99, 1.09) | ref | 0.382 |
| Any CVD | 1.00 (0.96, 1.03) | 1.02 (0.98, 1.07) | 1.00 (0.96, 1.04) | ref | 0.624 |
| **Dietary Intake** |  |  |  |  |  |
| Consumed ≥ 1 veggie/day | 1.01 (0.98, 1.048) | 1.01 (0.96, 1.06) | 1.04 (1.00, 1.08) | ref | 0.331 |
| Consumed ≥ 1 fruit/day | 0.97 (0.95, 1.004) | 0.97 (0.94, 1.02) | 1.01 (0.98, 1.05) | ref | **0.016** |
| Met fruit&veggie guideline^b^ | 0.97 (0.91, 1.044) | 1.00 (0.91, 1.11) | 0.99 (0.90, 1.09) | ref | 0.798 |
| Met sugary drink guideline^c^ | 0.93 (0.85, 1.011) | 0.97 (0.84, 1.13) | 0.96 (0.85, 1.09) | ref | 0.356 |
| **Physical Activity** |  |  |  |  |  |
| Any exercise | 1.01 (0.98, 1.04) | 1.01 (0.97, 1.04) | 1.02 (0.99, 1.06) | ref | 0.543 |
| Met aerobic guideline^d^ | 0.98 (0.96, 1.01) | 0.98 (0.94, 1.02) | 1.01 (0.98, 1.05) | ref | **0.105** |
| Met strengthening guideline^e^ | 0.99 (0.96, 1.03) | 0.95 (0.91, 1.00) | 0.98 (0.94, 1.03) | ref | 0.172 |
| Met both PA guidelines | 1.00 (0.96, 1.05) | 0.95 (0.90, 1.01) | 0.99 (0.95, 1.05) | ref | 0.115 |

^a^BMI ≥30 kg/m^2^

^b^fruit&veggie guideline: ≥4.5 times/day

^c^met sugary drink recommendation: ≤3 drinks/week

^d^met aerobic guideline: ≥150 min/week moderate-to-vigorous intensity physical activity

^e^met strengthening guideline: at least 2 times/week
